# Treatment of Granulomatous Inflammation in Pulmonary Sarcoidosis

**DOI:** 10.3390/jcm13030738

**Published:** 2024-01-27

**Authors:** Alicia K. Gerke

**Affiliations:** Pulmonary and Critical Care Medicine, University of Iowa, 200 Hawkins Drive, Iowa City, IA 52242, USA; alicia-gerke@uiowa.edu

**Keywords:** granuloma, treatment, immunosuppression, corticosteroids, sarcoidosis

## Abstract

The management of pulmonary sarcoidosis is a complex interplay of disease characteristics, the impact of medications, and patient preferences. Foremost, it is important to weigh the risk of anti-granulomatous treatment with the benefits of lung preservation and improvement in quality of life. Because of its high spontaneous resolution rate, pulmonary sarcoidosis should only be treated in cases of significant symptoms due to granulomatous inflammation, lung function decline, or substantial inflammation on imaging that can lead to irreversible fibrosis. The longstanding basis of treatment has historically been corticosteroid therapy for the control of granulomatous inflammation. However, several corticosteroid-sparing options have increasing evidence for use in refractory disease, inability to taper steroids to an acceptable dose, or in those with toxicity to corticosteroids. Treatment of sarcoidosis should be individualized for each patient due to the heterogeneity of the clinical course, comorbid conditions, response to therapy, and tolerance of medication side effects.

## 1. Introduction

Sarcoidosis is a multi-system disease of granulomatous inflammation that affects the lungs in the vast majority of those afflicted. Spontaneous resolution occurs in over half of patients, but the rest can develop chronic symptoms, progressive organ dysfunction, or a waxing and waning course. Treatment of inflammation is the basis of therapy; however, management decisions are often complex due to the variability of clinical course, differing patient responses to therapies, and uncertainty regarding the dose and duration of medications. In the current concept of sarcoidosis management, treatment is suppressive rather than curative. Herein, the mechanisms of anti-granulomatous therapy for sarcoidosis, practical clinical applications, and future directions of sarcoidosis management are reviewed.

## 2. Pathophysiology

The hallmark of sarcoidosis is the formation of nonnecrotizing granulomas. The amplified immune response is presumably due to an environmental antigen in a genetically predisposed individual. The concept of an inciting exposure is supported epidemiologically by the association of a higher incidence of sarcoidosis with occupational (e.g., firefighters, agricultural jobs, and Navy personnel) and environmental exposures (e.g., insecticides and mold/mildew exposures), as well as case cluster events such as the World Trade Center collapse [1,2,3]. Familial clusters of sarcoidosis, the identification of gene variants associated with disease development, and differences in prevalence rates globally suggest a genetic component [4,5]. Genome-wide association (GWA) scans of both familial and sporadic sarcoidosis incident cases have identified numerous chromosomal regions, particularly in the major histocompatibility complex (MHC) locus, contributing to sarcoidosis risk; however, no single gene has been identified as a main contributor [6]. Gene-environment interactions influencing phenotype have further been noted, such as the DRB1*11:01 gene interaction with mold and musty odors in the development of pulmonary sarcoidosis [7]. Ideally, definitive treatment of sarcoidosis would involve the removal of the antigen to cease the inflammatory response and cure the disease. However, it is possible that the antigen will remain unknown or differ for each specific case. Until the antigens are identified, current treatment strategies target the steps of the cascade that initiates and perpetuates granulomatous inflammation.

**Figure 1:**
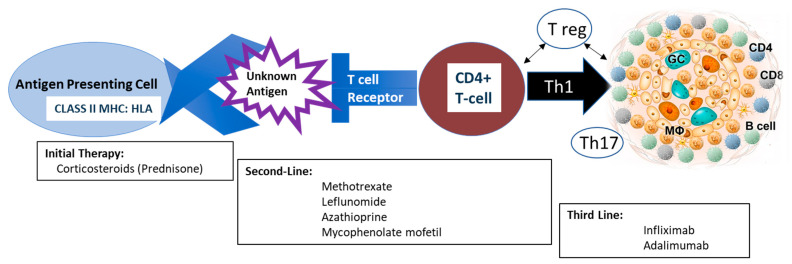
Pathophysiology of granulomatous inflammation in sarcoidosis and medications utilized in treatment. The granuloma of sarcoidosis is a tightly formed conglomeration of macrophages (Mθ) and multinucleated giant cells (GC), surrounded by a well-formed ring of CD4+ T cells interspersed with CD8+ T cells and rare B cells. Initiation of the immune response is thought to be due to the presentation of an unknown antigen that triggers the production of numerous cytokines and chemokines, which then attract cells of the adaptive immune response, resulting in a highly polarized Th1 response. Activated lymphocytes, macrophages, and mononuclear cells then migrate to the lung, forming granulomas. Both regulatory T cells and TH17 responses are also involved, although less well-delineated than the Th1 response.

The granuloma of sarcoidosis is a tightly formed conglomeration of macrophages and multinucleated giant cells, surrounded by a well-formed ring of CD4+ T cells interspersed with CD8+ T cells and an occasional B-cell [8]. The initiation of the immune response is thought to be due to the presentation of the antigen via the MHC complex, which triggers the production of numerous cytokines and chemokines, which then attract cells of the adaptive immune response. Dendritic cells likely play an important role in the presentation of antigen and continued immune response, although there are few studies establishing the exact role of these cells [9]. The abundance of current data indicates that sarcoidosis is a highly polarized Th1 response with a predominance of CD4+ lymphocytes and fewer CD8+ lymphocytes producing interferon-gamma [10]. Activated lymphocytes, macrophages, and mononuclear cells then migrate to the site of granulomatous inflammation in a process driven by the amplification of oligoclonal T-cells, forming granulomas (Figure 1). The cells within the granulomas in the lungs appear to be the result of both in situ proliferation and redistribution of cells from peripheral blood. Importantly, the cells involved in this immune response, as well as many of the upregulated cytokines, are targeted by current therapies with the goal of breaking the immunologic cycle of activation. Dysfunction of regulatory T-cells (“T regs”) and immune ‘exhaustion’ with failure to clear an antigenic agent may also play a role in the lack of immune resolution, which has opened discussion regarding alternative future medication targets [11].

## 3. When to Treat

Because over half of patients will spontaneously resolve, treatment is not indicated for patients with asymptomatic disease. For example, in patients with pulmonary sarcoidosis, mediastinal and hilar lymphadenopathy is rarely symptomatic and is not usually an indication to treat. In many cases, a period of close monitoring is warranted prior to the initiation of therapy to determine the disease course. In the setting of progressive disease, which is determined by lung function decline, significant symptoms, or worsening radiographic findings, treatment is indicated to preserve lung function and improve quality of life [12]. Patients with mild symptoms may be monitored unless radiographic findings suggest imminent danger of organ dysfunction (e.g., computed tomography (CT) findings of moderate to severe parenchymal lung disease). More prominent symptoms often include dyspnea, cough, fatigue, and atypical chest pain. Objective and subjective assessments may not always correlate, making patient-centered discussions regarding risks and benefits important prior to the initiation of anti-inflammatory therapy. Similarly, even concurrent objective measurements such as pulmonary function and the severity of CT chest imaging may not correlate, complicating disease assessment [13]. Thus, the decision to treat is rarely straight-forward. Ultimately, the goals of anti-inflammatory treatment are two-fold: 1. To alleviate debilitating symptoms that impair quality of life; and 2. To preserve organ function and prevent fibrosis by decreasing repetitive inflammation, tissue injury, and aberrant healing. The benefits of treatment must outweigh the likely toxicity of medications.

## 4. Medications Targeting Granulomatous Inflammation

*Corticosteroids:* Corticosteroids have long been the basis of first-line treatment for sarcoidosis, as they are very effective at immune suppression [14,15]. Corticosteroids act to repress many genes responsible for the cytokine cascade that perpetuates the Th1 inflammatory response, including those responsible for the production of interleukin (IL)-1 and TNF-α [16]. In cases of progressive lung dysfunction and significant symptoms, corticosteroids have been shown to improve biomarkers, symptoms, chest X-ray scores, and spirometry in the short term (up to two years) [17]. Data beyond that are lacking, particularly regarding mortality benefits or modifications of the natural history. The exact immunosuppressive treatment regimen to prevent fibrosis is unknown, and initial treatment considerations are similar for non-fibrotic sarcoidosis and for fibrotic pulmonary sarcoidosis with concurrent inflammation. Establishing long-term efficacy is difficult as corticosteroid therapy is the standard of care, and withholding treatment or the use of placebo in cases of organ damage is deemed unethical by most treating physicians. Whether a stepwise approach to medications or more aggressive initial therapy is superior warrants further study.

Prednisone is the most commonly used corticosteroid for the treatment of sarcoidosis. Prednisone is metabolized rapidly in the body to prednisolone, which is responsible for the anti-inflammatory effects. Patients with severe liver disease may have impaired this process, affecting drug efficacy. The initial recommended dose for pulmonary sarcoidosis is 20 to 40 mg per day, with the goal of tapering down to the lowest dose that provides the maximum benefit and minimizes the side effects [12]. Prednisone is readily bioavailable for most patients and rapidly absorbed, although because of the metabolism via the cytochrome P450 3A4 pathway, drug interactions should be considered in the dosing decision. Varying tapering schedules have been proposed, but there are no standardized guides. Exact dosing regimens differ, in part, due to the variability in severity and clinical course for each individual patient.

For pulmonary sarcoidosis and most other manifestations, the estimated dose of 20 to 40 mg is a reasonable dosing range for efficacy. A Delphi study of treating physicians showed consensus among experts that doses above 40 mg provide no additional benefit [18]. Similarly, a study on the treatment of cardiac sarcoidosis in Japanese patients showed those who were treated with higher doses of corticosteroids (greater than 40 mg per day) compared to lower doses (less than 30 mg per day) had higher morbidity and mortality, with no additional clinical benefit [19]. In a longer-term follow-up study of patients with pulmonary sarcoidosis, the initial starting dose of prednisone was not associated with greater improvement in lung function, although the side effect of weight gain was significant in those starting at higher doses [20]. It is important to note, however, that higher initiating doses may be necessary in cases of severe extrapulmonary involvement with life-threatening or organ-threatening manifestations such as neurologic demise or blindness.

The tapering of corticosteroids can be individualized based on response. In pulmonary sarcoidosis, the lung function improvement is seen within the first month of treatment and maximized by three months, arguing for early re-evaluation after treatment initiation [21]. A few proposed tapering regimens have been published, but there are no head-to-head studies. Conceptually, corticosteroid dosing includes a higher-dose initiation phase, decreasing the dose to a tolerable maintenance dosage of 7.5 mg to 15 mg per day, then tapering off therapy. A Delphi consensus suggests that sarcoidosis experts generally agree that maintenance doses above 10 mg per day are not acceptable [18]. Treatment duration averages around one year, although a large British series reported long-term success after five years (as determined by improved lung function) with an eighteen-month tapering regimen [22], whereas others have proposed shorter durations of even six months in some patients with favorable clinical courses [23].

Unfortunately, despite the efficacy of corticosteroids for immunosuppression, long-term use has significant toxicity that is dose- and duration-dependent and may even affect outcomes adversely. A large meta-analysis of mortality in sarcoidosis showed that patients treated in referral centers were seven times more likely to receive corticosteroids and had a higher mortality rate (4.8%) compared to those from population-based centers (0.5%), which could not be accounted for by stage of disease or ethnicity [24]. Higher cumulative doses are associated with poor quality of life and increased emergency room visits in patients with sarcoidosis [25]. Even cumulative low doses or periodic dosing have been shown to have adverse effects for many chronic inflammatory diseases [26,27]. For the duration often needed to treat sarcoidosis, clinicians must be aware of side effects that can complicate management (Table 1). Furthermore, many of the side effects of prednisone, or symptoms due to the withdrawal of prednisone, can mimic nonspecific symptoms of sarcoidosis, such as weakness, fatigue, and dyspnea, confounding management decisions. For these reasons, clinicians often consider corticosteroid-sparing options to alleviate the burden of disease.

Of note, inhaled corticosteroids are occasionally used in the treatment of pulmonary sarcoidosis, despite the lack of positive randomized controlled trials (RCTs) [28]. Inflammation frequently affects the airways of patients with sarcoidosis, and inhaled methods have few side effects with theoretical benefit, making them an attractive option for clinicians. Older pilot studies suggested a benefit [29,30,31], but most subsequent RCTs failed to show objective effects, although many of these patients were concurrently treated with oral therapy [32,33]. One study from the Dutch Study Group on Pulmonary Sarcoidosis did find an improvement in symptoms (cough) and inspiratory vital capacity, but no change in serum ACE, diffusing capacity, or chest imaging [34]. Future studies are needed to delineate the anti-inflammatory effect of inhaled therapy and the exact type of patient who may benefit.

*Corticosteroid-Sparing Medications*: The long-term use of corticosteroids is still the standard treatment for sarcoidosis. This contrasts with the treatment of other autoinflammatory and autoimmune diseases, including rheumatoid arthritis and inflammatory bowel disease, where the early use of a steroid-sparing agent is well established. This may be due to the paucity of clinical trials with these agents in sarcoidosis. A systematic review from 2010 summarized immunosuppressive and cytotoxic therapy for pulmonary sarcoidosis and concluded that the current body of evidence supporting the use of cytotoxic therapies is limited [35]. However, increasing data from the last decade supports the role of corticosteroid-sparing agents in many cases of chronic or progressive sarcoidosis. Certain older medications, such as cyclosporine, chloroquine, and cyclophosphamide, have fallen out of favor due to their severe side effects and are not currently recommended for sarcoidosis in general. Some therapies are more organ-specific, such as hydroxychloroquine. It is useful for skin involvement or hypercalcemia, but it does not seem to be as effective for lung involvement. On the other hand, there are efficacious and well-tolerated drugs that are employed in the treatment of pulmonary sarcoidosis to minimize the toxicity of long-term corticosteroid use. Additionally, it can be argued that, in cases with poor prognostic factors or severe organ risk, aggressive treatment and early control of inflammation may be of value in improving outcomes [36].

The timing of second-line agents is debatable and can be affected by several factors, including comorbid conditions, cost, patient preference, tolerance of side effects, and the characteristics of the inflammatory response itself. Generally, indications for a steroid-sparing agent include refractory disease, inability to taper corticosteroids, or intolerance/toxicity to corticosteroid therapy [15]. Interestingly, corticosteroid resistance in patients with sarcoidosis has been associated with exaggerated TNF-α release by alveolar macrophages, arguing for the potential use of alternative therapies (such as a TNF antagonist) to augment response and decrease steroid dose [37]. As noted above, most clinicians feel that corticosteroids above 10 mg per day for any phenotype would be an acceptable reason to initiate a second-line agent [18]. Given the long half-lives of many of the corticosteroid-sparing options, tapering corticosteroids is reasonable 1 to 2 months after the initiation of the second-line agent. In ideal situations, corticosteroids can be tapered off completely and replaced; however, in some cases, dual therapy is necessary for the best effect. Future studies are warranted to determine the efficacy of up-front monotherapy with a corticosteroid-sparing medication, combination therapy with corticosteroids, and as a replacement agent, as is often seen in other diseases such as rheumatoid arthritis or inflammatory bowel disease [38,39].

Methotrexate is the most recommended corticosteroid-sparing medication, supported by studies showing improvement in forced vital capacity and symptoms when used as a steroid-sparing agent [15,40,41]. Methotrexate is an anti-metabolite with a myriad of actions upon the immune response via folate antagonism and through adenosine pathways that suppress inflammatory cytokine production by monocytes and macrophages [42]. Its efficacy is not universal (55–80%), however, and may depend on pharmacogenetic profiles or dose-limiting side effects [42,43]. Generally, dosing ranges from 7.5 mg to 15 mg per week, depending on age, weight, and renal function, but higher doses, such as 20 to 25 mg per week, can be used at times if there is an inadequate response [40]. It can take up to six months for full efficacy. Methotrexate is usually started at a low dose and increased every 2 to 4 weeks to the goal dose. It can be used subcutaneously in the same doses in patients with gastrointestinal side effects or concern for absorption. Folic acid is used in conjunction with methotrexate to reduce toxicity from gastrointestinal distress, transaminitis, and mouth ulcerations to help maintain compliance [44]. Regular testing of liver function, blood counts (to assess for neutropenia), and renal function should be carried out while on methotrexate. Rare cases of pulmonary toxicity can occur and should be considered with an unexplained worsening of lung infiltrates and symptoms. Methotrexate is being studied as a first-line agent compared with prednisone in an ongoing clinical study [45].

Azathioprine, an inhibitor of purine and protein synthesis in lymphocytes, likely has equal efficacy to methotrexate as a second-line agent in terms of lung function improvement and the ability to taper steroids [46,47,48]. However, in a large retrospective cohort, a potential for increased risk of infection was seen in patients treated with azathioprine as compared to methotrexate [46]. The ideal dose for sarcoidosis is not known but is usually started at 50 mg per day and titrated up to effect in a 1 to 2 mg/kg/day dosing range, with a maximum dosage of 200 mg/day. Complete blood counts and liver function are monitored regularly.

Leflunomide, another anti-metabolite that inhibits dividing lymphocytes and promotes the T-reg response, has shown efficacy in case series. It can be used as an alternative to, or in combination with, methotrexate [49,50]. In one larger series of 76 patients with progressive sarcoidosis or those who failed other second-line agents, effects were seen in forced vital capacity and in extrapulmonary disease [49]. Leflunomide has the same monitoring requirements as methotrexate due to similar side effects, including liver toxicity, GI distress, pulmonary toxicity, and peripheral neuropathy. Dosing is 20 mg/day, although 10 mg/day doses can be trialed during initiation if tolerance is a concern. If toxicity is of immediate concern, cholestyramine can be used to bind and remove the drug from the body more rapidly.

Mycophenolate mofetil, converted to mycophenolic acid upon ingestion, is another useful second-line agent that has been shown to be of benefit in several case series, mostly via its steroid-sparing effect [51,52]. Lung function improvement was not as obvious, although a greater effect was seen in patients who were intolerant rather than refractory to other therapies [51]. Mycophenolate mofetil inhibits purine nucleotide synthesis in lymphocytes, disrupts proliferation, and decreases the production of autoantibodies by B cells. Notably, mycophenolate tends to have a better tolerability profile than most other options for second-line treatment, but monitoring for leukopenia is still advised. Dosing ranges from 500 mg twice daily to 1500 mg twice daily, and an enterically coated option is available for patients with gastrointestinal side effects.

Third-line therapies are used in cases where patients are refractory to second-line agents or intolerant of all options. In one large referral center, 15% of patients received at least one third-line agent [53]. Third-line agents include the TNF antagonists infliximab and adalimumab. Infliximab, a chimeric monoclonal antibody given by intravenous (IV) infusion, have the strongest data supporting its use in pulmonary sarcoidosis [54,55,56,57]. Because of the large role that TNF-α plays in the proliferation of granulomatous inflammation, impeding the action of the cytokine would suggest a beneficial effect in the treatment of sarcoidosis. A double-blind RCT in 138 patients with chronic pulmonary sarcoidosis showed that treatment with infliximab increased FVC by 2.5%, whereas the placebo group did not improve lung function [54]. Further analysis showed benefits in surrogate measures such as measured serum cytokines and reticular opacities on chest X-rays. Although the improvement in lung function was statistically significant, its clinical impact was debated. However, increasing data from large case series supports its beneficial use in pulmonary sarcoidosis, particularly the imaging findings in pulmonary disease [56]. Dosing of infliximab is 5 mg/kg IV at weeks 0, 2 and every 4–8 weeks thereafter [58]. Low-dose corticosteroids or methotrexate are often co-administered to prevent antibody formation, albeit with an undetermined increased long-term risk of malignancy when combination therapy is used [59]. Adalimumab is a fully human monoclonal antibody to TNF-α. Its effect has particularly been noted in extrapulmonary disease (e.g., skin and ocular involvement), but small case series show effects in pulmonary disease and in those who have developed intolerance to infliximab [60,61,62,63,64,65]. The effects seen in current trials of anti-TNF therapy may be muted by the fact that most enrolled patients have been refractory to other agents. Adalimumab is a subcutaneous administration of 40 mg every two weeks, but the dose can vary depending on response. Interestingly, anti-TNF therapy can cause a sarcoid-like reaction, which can complicate management in rare cases [66]. Current limited data suggest similar efficacy with biosimilars for the TNF antagonists (with possible cost benefits), but further evaluation is necessary as clinical data for these drugs accumulates [67,68]. In the long term, the most common reasons for drug discontinuation for infliximab and adalimumab include allergic reactions, infections, insurance denial, and loss/lack of efficacy [53]. In less than 10% of patients, the drug is discontinued because remission is achieved [53].

Of note, pentoxifylline is an older oral medication that inhibits the production of TNF-α from macrophages. It is not commonly used, but some limited data suggests that it may have a mild steroid-sparing effect when used in conjunction with corticosteroids [69,70]. Etanercept, a TNF receptor antagonist, was shown to be ineffective for sarcoidosis, as were other biologics such as golimumab (anti-TNF) and ustekinumab (an antibody to IL-21/IL-23) [71,72].

*Other Therapies with Limited Data*: As insights into the pathophysiology of granulomatous inflammation emerge, both new and re-purposed drugs are being evaluated for use in pulmonary sarcoidosis. B-cells are known to be present in the periphery of the granuloma and may play a role in granulomatous inflammation or altered immune homeostasis that leads to non-resolving disease. For this reason, rituximab, an anti-CD20 monoclonal antibody that depletes B cells, has been proposed as an option in refractory disease. In a small prospective study of ten patients with refractory disease, seven patients responded to therapy either by a 5% increase in FVC or improvement in walk distance by at least 30 m [73]. However, two deaths in the group (likely related to progressive sarcoidosis) and concern about infection risk dampen excitement around the use of rituximab, except in rarer cases of severe refractory disease. Other drugs that have potential include the Janus kinase (JAK) inhibitor tofacitinib, which has shown both a steroid-sparing effect and improvement in imaging biomarkers in two small series that included pulmonary evaluation [74,75,76]. Similarly, tocilizumab, an anti-IL-6 antibody, showed a significant response in a series of four patients who were refractory to alternative medications [77]. Transdermal nicotine is also under current study based upon data from a RCT of thirteen patients showing nicotine treatment normalized toll-like receptor (TLR) 2 and 9 responses and increased the T regulatory response in patients with pulmonary sarcoidosis [78]. The potential immunomodulating effect is also supported by epidemiologic data showing that smoking is a protective factor in the development of sarcoidosis [1]. Repository corticotropin injection has garnered interest, with an early small RCT showing improvements in pulmonary function, quality of life parameters, imaging, and a steroid-sparing effect in patients with chronic sarcoidosis [79]. A more recent study was unable to reproduce these improvements statistically but was able to show a faster steroid-tapering effect than standard of care [80]. As with many past clinical trials, controversy exists over the true efficacy of many medications, as limitations of sample size and inclusion criteria often hamper drug development in this disease. These and other potential immune modulating agents approved for other rheumatologic and inflammatory diseases may be candidates for larger clinical studies to determine efficacy and target populations.

A novel biologic drug, efzofitimod, has recently been tested in a safety and tolerability study in 37 patients with pulmonary sarcoidosis [81]. Efzofitimod binds to the neuropilin-2 receptor protein and modulates the immune cells in the granulomatous reaction, decreasing inflammation and fibrosis in preclinical studies. Results showed no difference in adverse events compared to placebo, a subtle improved steroid-sparing effect, and statistically significant improvement in patient-reported outcomes (Sarcoidosis Assessment Tool, King’s Sarcoidosis Questionnaire (KSQ), Fatigue Assessment Scale, KSQ general health) at the higher dose range [82].

## 5. Clinical Considerations in the Choice of Corticosteroid-Sparing Therapies

In deciding on a corticosteroid-sparing agent, it is also important to consider alcohol use, fertility concerns, and the presence of extrapulmonary involvement. Comorbid conditions such as liver or kidney dysfunction may also sway the choice or dosage of therapy. Other comorbidities, such as uncontrolled diabetes, hypertension, or obesity, may preclude the use of corticosteroids in some cases. Medication interactions, even with commonly used drugs such as antibiotics and anticoagulants, can be extensive with the use of corticosteroid-sparing medications and should be reviewed prior to initiation and when any new drug is prescribed. For example, methotrexate is contraindicated with concurrent alcohol use and should not be used by those desiring pregnancy or with inadequate birth control. Methotrexate should also be avoided in those with significant liver disease, a low glomerular filtration rate (less than 30 mL/min), and may need dose adjustment for those with mildly impaired renal function. Infliximab should only be used with great caution after consultation with a cardiologist in patients with heart failure. 

**Figure 2:**
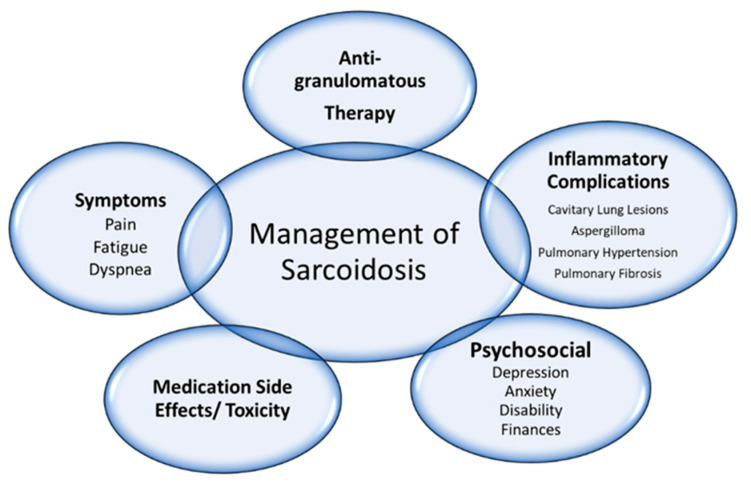
Complexity of management in pulmonary sarcoidosis. The treatment of a patient with sarcoidosis requires consideration of several aspects of care, not only treatment of granulomatous inflammation.

When initiating any type of anti-granulomatous therapy, detailed preparation and counseling of the patient are important to manage and alleviate toxicity. Prior to therapy, evaluation for hepatitis, tuberculosis, endemic fungi, and HIV should be considered. Vaccinations should be given, if possible, prior to therapy and updated periodically per guideline recommendations [83]. Medications may be held during times of infection or perioperatively. *Pneumocystis jirovecii* prophylaxis should be considered for patients on higher-dose corticosteroids for a prolonged period, particularly if combined with a steroid-sparing agent. Routine lab assessments, including complete blood counts and comprehensive metabolic panels specific to each drug, should be obtained and monitored closely for drug toxicity [84,85,86]. Shared decision-making with a patient and clinician is important to incorporate compliance barriers (both external and internal), risk acceptance, and patient preferences into the choice of drug. In the setting of progressive fibrotic lung disease despite anti-granulomatous therapy, clinicians may consider anti-fibrotic medication (not further discussed within this review) and should be referred for lung transplant evaluation if no other contraindications exist [87,88]. Additionally, it is important to address other aspects of care associated with sarcoidosis, including depression, anxiety, pain, and fatigue, which are not treated with anti-granulomatous therapy (Figure 2).

Pharmacogenetics may also play a role in the efficacy and tolerability of treatment options for sarcoidosis. Glucocorticoid receptor gene polymorphisms may affect sensitivity and response to glucocorticoids, thereby affecting dosing. Similarly, the lack of response to methotrexate in up to one-third of patients may be influenced by several potential polymorphisms in genes that are involved in the metabolism of methotrexate [89]. However, given the complexity of methotrexate genetics and metabolism, genetics are not used in the clinical realm for this medication. Azathioprine metabolism is also affected by mutations in the gene coding for thiopurine S-methyltransferase (TPMT), increasing the risk of toxicity, particularly in leukopenia [90]. TPMT enzyme activity with phenotyping can be measured in clinical practice. For infliximab, TNF-α polymorphisms within the TNF-α G-308A gene have been shown to predict response [91].

## 6. Relapses

Current recommendations to treat for approximately a year are based on a high relapse rate associated with shorter courses of therapy. A ‘relapse’ of sarcoidosis is based upon a significant need to increase systemic anti-inflammatory medications and worsen dyspnea, chest imaging, and pulmonary function [92], with chronic sarcoidosis cases having higher rates of relapse. Most relapses are seen within six months of cessation of therapy, but approximately 20% of relapses occur after one year, indicating the need for long-term monitoring after treatment [93]. It is unclear if this relapse rate is affected by the type of initial therapy. Some prior data have suggested a higher relapse rate in those treated only with corticosteroids [93]; how this varies with different combinations of immunomodulating therapies is unknown. In one study of advanced sarcoidosis cases requiring third-line therapies, approximately half of patients had to discontinue drugs, and 50–93% had recurrences requiring re-initiation of therapy [53]. Treatment of relapses mimics the original successful doses, although one study suggested that lower dosing regiments may be just as effective for relapses [94]. Alternatively, relapses often indicate a prolonged, chronic course, broaching the benefits of an early corticosteroid-sparing agent in longer-term management [95]. Predicting relapses is difficult, although recent data have suggested that high Fluorodeoxyglucose (FDG)-PET uptake in the lung is associated with relapse after cessation of therapy, including after corticosteroids and infliximab [96,97]. Similarly, high levels of soluble interleukin-2 receptor (sIL-2R) have also been associated with relapse after treatment with infliximab, making it an intriguing marker of prognosis once a medication has been stopped [96].

## 7. Biomarkers in the Management of Therapy

Biomarkers have been an active area of study in sarcoidosis management, although standardized diagnostic and prognostic markers are still lacking. Advanced imaging techniques have shown promise in the past decade that may be useful in the prognosis and evaluation of treatment efficacy. FDG-PET/CT scans have been used to evaluate granulomatous inflammation and can be used to identify sites of reversible activity as well as clinical response. Positive uptake in FDG-PET is seen in patients with radiographic stages 2 and 3 sarcoidosis, whereas negative uptake is common in stages 0, 1, and 4 [98]. Its use has been suggested as a potential marker to differentiate the presence of potential reversible inflammatory disease-requiring therapy or irreversible fibrosis in those with advanced lung disease, but its use in this manner is controversial [99,100]. Because the clinical utility and cost-effectiveness of FDG-PET remain unclear, it is not routinely used in the diagnosis and management of pulmonary sarcoidosis. However, it could potentially be useful in combination with other biomarkers in difficult cases.

Serum biomarkers are also potentially useful in treatment assessment [101]. Angiotensin-converting enzyme does modestly correlate with parenchymal burden in the lung. High levels of ACE and sIL-2, another biomarker of the Th1 inflammatory cascade, are associated with improvement in lung function after six months of treatment with methotrexate [102]. Subtyping of bronchoalveolar lavage cells could also be informative for pulmonary sarcoidosis, as the cells are a direct window to the lung microenvironment. Higher neutrophil counts have been linked to a lower response to therapy [103]. More recently, the presence of Th17 cells has been associated with the development of chronic sarcoidosis, and the ratio of Th1 cells and T cells also holds promise in prognostication [104,105].

Genomics has also been revealing in both diagnostic and prognostic biomarker evaluation. A recent meta-analysis of transcriptome-wide association studies of tissue developed a prediction classifier using gene expression profiles that could discern sarcoidosis from healthy controls in the lymph nodes [106]. Similarly, based on the evaluation of candidate genes identified in genomic analysis, plasma biomarkers extracellular nicotinamide phosphoribosyl transferase (eNAMPT) and angiopoietin-2 (ANG-2) have also been associated with complicated phenotypes and pulmonary fibrosis [107]. The finding of certain genes and proteins associated with fibrosis or the discernment of progressive phenotypes may, in the future, be an intriguing way to determine the need for and type of treatment.

Early work in the field of artificial intelligence also holds promise for future prognostic and management biomarkers, although it is currently predominately focused on diagnosis [108]. Radiomics, the interpretation of imaging characteristics not seen by the human eye, has been able to help differentiate sarcoidosis from malignancy and other granulomatous diseases. Certain measures have been correlated to pulmonary function testing, which is notable given the historical discrepancy in pulmonary function and qualitative imaging [109]. Machine and deep learning methodologies have also been used to create a decision tool for the diagnosis of sarcoidosis from imaging data and to help differentiate pulmonary sarcoidosis from tuberculosis [110,111]. As further validation of these techniques evolves, their application as novel outcome measures and prognostic biomarkers holds great promise, particularly as larger datasets incorporate serum, tissue, imaging, and clinical data.

## 8. Summary

Management of pulmonary sarcoidosis is a complex interplay of disease characteristics, the impact of medications, and patient preferences (Figure 2). First, it is important to weigh the need for anti-granulomatous treatment with the risks of toxicity that each treatment entails. Pulmonary sarcoidosis should be treated in cases of significant symptoms, lung function decline, or progressive pulmonary inflammation that poses a risk to the lung. The basis of treatment is corticosteroid therapy for initial control of granulomatous inflammation, but corticosteroid-sparing agents can be initiated in cases of refractory disease or toxicity to corticosteroids. Each case of sarcoidosis will have an individualized plan due to the heterogeneity of the clinical course, response to therapy, and tolerance of medication side effects.

## 9. Future Directions

Future directions in anti-granulomatous treatment in pulmonary sarcoidosis include clinical trials based on careful phenotyping to establish the long-term efficacy of anti-inflammatory medications and whether these therapies prevent pulmonary fibrosis. Additionally, trials of dual therapy, duration and dosing of therapy, or steroid-sparing monotherapy would aid in diminishing the corticosteroid side effect profile and establishing standardized treatment guidelines. Head-to-head trials of anti-metabolites may also reveal the order and choice of medications, and novel formulations of repurposed drugs that minimize toxicity would be helpful. The development of home monitoring devices, such as reliable spirometry or symptom reporting tools, could also aid in accelerated steroid-tapering.

In this manner, there is much work to be carried out in clinical trial development for pulmonary sarcoidosis to enroll the necessary population and develop better outcomes that show the important effects of drug therapy. New methods within radiomics, genomics, and proteomics, possibly aided by artificial intelligence, could help with more accurate phenotyping of patients. Additionally, novel drug development based on emerging knowledge of the pathophysiology of disease that will diminish the toxicity of treatment could revolutionize treatment paradigms. Ultimately, the discovery of the cause of sarcoidosis will lead to the cure and prevention of disease in the future.

## Figures and Tables

**Table 1:** Management of common side effects and toxicity of corticosteroid use in sarcoidosis.

Corticosteroid Toxicity	Clinical Considerations
Suppression of the hypothalamic-pituitary-adrenal axis	Close attention to the tapering of corticosteroids. Differentiation of withdrawal symptoms versus sarcoidosis.
Increased susceptibility to infection	Evaluation of new cough, sputum, and dyspnea to differentiate from sarcoidosis. Administer vaccines prior to immunosuppression. At high doses, *Pneumocystis jirovecci* prophylaxis.
Weight gain	Counseling on appetite side effects, dietary management, and exercise program.
Ocular complications: cataracts, glaucoma	Routine eye exams.
Impaired bone density	Consider concurrent bisphosphonates, bone density scans, and exercise.
Steroid-induced myopathy	Consider continued tapering of the dose and an exercise program.
Dermatologic effects: skin thinning, bruising, acne, Cushingoid features	Consider a dermatology referral. Wean corticosteroids to the lowest possible dose.
Fluid retention	Caution in heart failure. May need the diuretic dose adjusted.
Hypertension	Blood pressure monitoring.
Gastric irritation and ulcer disease	Consider concurrent acid-suppression therapy.
Hyperglycemia	Caution in diabetes: monitor blood glucose closely.
Insomnia, Dysthymia, and Psychosis	Counsel the patient about potential psychiatric and neurologic effects.
